# Supplementary material for: Prion propagation can occur in a prokaryote and requires the ClpB chaperone
Source: eLife. 2014 Aug 13;3:e02949. doi: 10.7554/eLife.02949 (PMC4150125; doi:10.7554/eLife.02949)
Supplement: Supplementary file 1. — Strains and Plasmids. DOI: http://dx.doi.org/10.7554/eLife.02949.017 [file elife02949s001.pdf]

Supplementary file 1

**Strains (*Escherichia coli*)**	**Alias**	**Genotype**	**Source and Comments**
DH5αZ1	AY75	*attB*(λ)*::lacI*^{q}-P$_{N25}$-*tetR::aadA endA1 hsdR17*(*rK-mK+*) *glnV44 thi-1 recA1 gyrA96 relA1* Δ(*lacIZYA-argF*)*169 deoR attB*(ϕ80)::*lacZ*Δ*M15*	Lutz and Bujard, 1997; Spec^{R}
DH5αZ1 Δ*clpB*	AY153	DH5αZ1 Δ*clpB*::*kan*	This study; Spec^{R} and Kan^{R}
PIR2 F' P$_{tac}$-*tetR*	AY290	Δ*lac169 rpo*S(*am*) *robA1 creC510 hsdR514 endA recA1 uidA(*Δ*MluI)*::*pir* F' P$_{tac}$-*tetR-lacZYAI*^{q}::*tetRA*	This study; Tet^{R}
DH5αZ1 Δ*clpB*	AY295	DH5αZ1 Δ*clpB*::*FRT*	This study
DH5αZ1 Δ*clpB* P$_{LtetO-I}$-*clpB*	AY321	DH5αZ1 Δ*clpB*::*FRT attB*(HK022)::P$_{LtetO-I}$-*clpB*-t$_{L3}$::*kan*	This study; Spec^{R} and Kan^{R}
DH5αZ1 Δ*clpB* P$_{LtetO-I}$-*clpB Y653A*	AY322	DH5αZ1 Δ*clpB*::*FRT attB*(HK022)::P$_{LtetO-I}$-*clpB Y653A*-t$_{L3}$::*kan*	This study; Spec^{R} and Kan^{R}
DH5αZ1 Δ*clpB* P$_{LtetO-I}$-*clpB E279A/E678A*	AY323	DH5αZ1 Δ*clpB*::*FRT attB*(HK022)::P$_{LtetO-I}$-*clpB E279A/E678A*-t$_{L3}$::*kan*	This study; Spec^{R} and Kan^{R}
DH5αZ1 Δ*clpB* P$_{LtetO-I}$-*clpB E432A*	AY324	DH5αZ1 Δ*clpB*::*FRT attB*(HK022)::P$_{LtetO-I}$-*clpB E432A*-t$_{L3}$::*kan*	This study; Spec^{R} and Kan^{R}
DH5αZ1 Δ*clpB* P$_{LtetO-I}$	AY325	DH5αZ1 Δ*clpB*::*FRT attB*(HK022)::P$_{LtetO-I}$-t$_{L3}$::*kan*	This study; Spec^{R} and Kan^{R}
**Strains (*Saccharomyces cerevisiae*)**	**Alias**	**Genotype**	**Source and Comments**
YJW187 [*pin*$^{-}$] [*psi*$^{-}$]	SG775	74D-694 *MATa ade1-14*(UGA) *his3 leu2 trp1 ura3* [*pin*$^{-}$] [*psi*$^{-}$]	Garrity et al., 2010; obtained by serial passage on YPD agar supplemented with 3 mM GuHCl; phenotypically [*pin*$^{-}$] [*psi*$^{-}$]
YJW187 [*PSI*$^{+}$]	SG862	74D-694 *MATa ade1-14*(UGA) *his3 leu2 trp1 ura3* [*PSI*$^{+}$]	Garrity et al., 2010; phenotypically strong [*PSI*$^{+}$]
YJW187 [*PSI*$^{+}$]	SG863	74D-694 *MATa ade1-14*(UGA) *his3 leu2 trp1 ura3* [*PSI*$^{+}$]	Garrity et al., 2010; phenotypically weak [*PSI*$^{+}$]
**Plasmids**	**Alias**	**Features**	**Source and Comments**
pBR322-*SUP35 NM*	pAY45	*bla* P$_{tac}$-*SUP35 NM-mCherry-his$_{6X}$*-t$_{T7}$ pMB1 *ori*	This study; produces Sup35 NM (Sup35 residues 1-253) fused to

Supplementary file 1

			mCherry2 and a hexahistidine tag under the control of the tac promoter; Amp^R
$pSC101^{TS}$	pAY50	*cat* P_{tac}-t_{L3} $repA^{TS}$ pSC101 *ori*	This study; derived from pMAK700 (Hamilton et al., 1989); Cam^R
$pSC101^{TS}$-*NEW1*	pAY48	*cat* P_{tac}-*NEW1-mGFP*-t_{L3} $repA^{TS}$ pSC101 *ori*	This study; produces residues 50-100 of New1 fused to $mGFP_{mut3}$ under the control of the tac promoter; Cam^R
$pSC101^{TS}$-*NEW1-clpB*	pAY71	*cat* P_{tac}-*NEW1-mGFP*-t_{L3}-P_{clpB}-*clpB* $repA^{TS}$ pSC101 *ori*	This study; produces residues 50-100 of New1 fused to $mGFP_{mut3}$ under the control of the tac promoter; produces ClpB under the control of its native promoter; Cam^R
$pSC101^{TS}$-*recA*	pAY69	*cat* P_{tac}-*recA*-t_{L3} $repA^{TS}$ pSC101 *ori*	This study; produces RecA under the control of the tac promoter; Cam^R
pAH70-$P_{LtetO-I}$	pAY152	*kan* $P_{LtetO-I}$-t_{L3} *attP*(HK022) γR6K *ori*	This study; derived from pAH70 (Haldimann and Wanner, 2001); harbors the $P_{LtetO-I}$ promoter(Lutz and Bujard, 1997); Kan^R
pAH70-$P_{LtetO-I}$-*clpB*	pAY154	*kan* $P_{LtetO-I}$-*clpB*-t_{L3} *attP*(HK022) γR6K *ori*	This study; produces wild-type ClpB under the control of the $P_{LtetO-I}$ promoter; Kan^R
pAH70-$P_{LtetO-I}$-*clpB Y653A*	pAY155	*kan* $P_{LtetO-I}$-*clpB Y653A*-t_{L3} *attP*(HK022) γR6K *ori*	This study; produces ClpB Y653A under the control of the $P_{LtetO-I}$ promoter; Kan^R
pAH70-$P_{LtetO-I}$-*clpB E279A/E678A*	pAY156	*kan* $P_{LtetO-I}$-*clpB E279A/E678A*-t_{L3} *attP*(HK022) γR6K *ori*	This study; produces ClpB E279A/E678A under the control of the $P_{LtetO-I}$ promoter; Kan^R
pAH70-$P_{LtetO-I}$-*clpB E432A*	pAY157	*kan* $P_{LtetO-I}$-*clpB E432A*-t_{L3} *attP*(HK022) γR6K *ori*	This study; produces ClpB E432A under the control of the $P_{LtetO-I}$ promoter; Kan^R
pAH69	*N/A*	*bla* λP_R-int_{HK022} λ*cI857* $repA^{TS}$ R101 *ori*	Haldimann and Wanner, 2001; produced HK022 Int under the control of the λP_R promoter; Amp^R
pCP20	*N/A*	*bla cat* λP_R-*flp* λ*cI857* $repA^{TS}$ p15A *ori*	Cherepanov and Wackernagel, 1995; produced Flp recombinase under the control of the λP_R promoter; Amp^R and Cam^R
pRS316	*N/A*	*URA3 bla CEN6* pMB1 *ori*	Sikorski and Hieter, 1989; *URA3* shuttle vector
